# Die Wirkung von elektromagnetischen Feldern auf Tendinopathien: Studie zur Effektanalyse einer singulären Anwendung von hochenergetischen gepulsten elektromagnetischen Feldern

**DOI:** 10.1007/s00132-024-04541-3

**Published:** 2024-08-22

**Authors:** Patrick Wilms, Jan Schröder, Lorenz Scheit, Rüdiger Reer

**Affiliations:** 1 Institut für Bewegungswissenschaft, Sport- und Bewegungsmedizin, Universität Hamburg, Turmweg 2, 20148 Hamburg, Deutschland; 2 Abteilung I – Innere Medizin, Bundeswehrkrankenhaus Hamburg, Lesserstr. 180, 22049 Hamburg, Deutschland

**Keywords:** Magnetfeldtherapie, Tendinopathie, Schmerz, Druckschmerzschwelle, HSP70, Magnetic field therapy, Tendinopathy, Pain, Pain pressure threshold, HSP70

## Abstract

**Zielsetzung:**

Tendinopathien sind Erkrankungen, die häufig eine langfristige Behandlung mit Analgetika, Physiotherapie, Orthesen und Schonung erfordern. Ziel dieser Studie war es, die Wirkung einer einmaligen Anwendung von hochenergetischem PEMF (pulsed electromagnetic field) auf das Schmerzempfinden und unspezifische Entzündungsparameter zu untersuchen.

**Methode:**

34 Patienten wurden nach dem Zufallsprinzip einer Verumgruppe (10 min PEMF; 0,78 T) oder einer Placebogruppe (10 min Scheinbehandlung) zugeteilt. Vor und bis zu einer Woche nach der patientenverblindeten Behandlung (t1–t5) wurde der lokale Schmerzzustand (Algometrie) als Druckschmerzschwelle (PPT, pain pressure threshold) ermittelt. Gleichzeitig wurden Hitzeschockprotein 70 (HSP70) Blutkonzentrationen analysiert. Das zweifaktorielle Datenmodell wurde varianzanalytisch ausgewertet (2-way ANOVA). Die Studie wurde als „clinical trial“ registriert (DRKS00031321).

**Ergebnisse:**

Nach Randomisierung und Drop-out (Verum *n* = 17, Placebo *n* = 13) ergaben Baseline-Analysen keine signifikanten Gruppenunterschiede für PPT (*p* = 0,096) oder HSP70 (*p* = 0,524), oder in Stichprobenmerkmalen (*p* > 0,05). Für die PEMF Gruppe zeigte sich ein signifikant stärkerer Anstieg (*p* = 0,045, η^2^ = 0,013) der Druckschmerzschwelle (PPT: +83 bis + 139 %) als für die Placebo Gruppe (PPT: +10 bis + 36 %). Für HSP70 zeigten sich keine assoziierten Effekte.

**Schlussfolgerungen:**

Eine einmalige Anwendung von hochenergetischem PEMF führte zu einer sofortigen Placebo-Effekt bereinigten Schmerzlinderung über eine Woche bei Tendinopathie-Patienten, aber der vermutete zugrunde liegende HSP70-assoziierte Entzündungsweg konnte nicht bestätigt werden.

**Graphic abstract:**

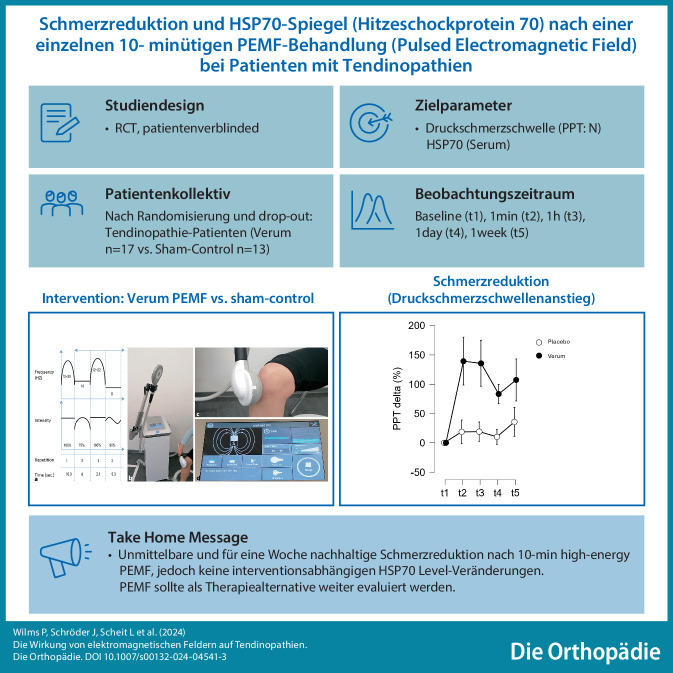

**Zusatzmaterial online:**

Zusätzliche Informationen sind in der Online-Version dieses Artikels (10.1007/s00132-024-04541-3) enthalten.

## Abstract (en)

**Objective:**

Tendinopathies are diseases that often entail long-term treatment consisting of analgesics, physiotherapy, orthotics, and sparing. The aim of this study was to investigate the effect of a single application of a high-energy PEMF (pulsed electromagnetic field) on pain perception and blood born inflammation parameters.

**Methods:**

34 patients were randomly assigned to a verum group (10 min PEMF, 0,78 T) or a placebo group (10 min sham condition). Prior to and up to one week after the patient blinded treatment (t1–t5), local pain state was assessed by means of algometry as pain pressure threshold (PPT). Accordingly, heat-shock protein 70 (HSP70) levels were analysed. Statistical analyses included 2‑way ANOVA (2 × 5). The clinical trial was registered (DRKS00031321).

**Results:**

After randomization and drop-out (verum *n* = 17, placebo *n* = 13) baseline-analyses did not reveal significant between-group differences for PPT (*p* = 0,096), for HSP70 (*p* = 0,524), or any other sample characteristics (*p* > 0,05). Pain reduction during one week of observation showed to be significantly higher (*p* = 0,045, η^2^ = 0,013) for the PEMF group (PPT: +83 bis +139%) compared to the placebo group (PPT: +10 bis +36%). There were no HSP70 associated effects.

**Conclusions:**

A single bout of high energy PEMF led to an immediate pain relief in tendinopathy patients lasting at least for one week, but the hypothesized underlying HSP70 associated inflammatory pathway could not be confirmed.

Tendinopathien sind Erkrankungen, die häufig eine langfristige Behandlung mit Schmerzmitteln, Physiotherapie, Orthesen und Schonung erfordern [1]. Die Ursache ist meist eine übermäßige mechanische Belastung. Dies führt zu einer Entzündung des Sehnenansatzes mit Veränderungen der Kollagenstruktur, der Vaskularisierung und der Freisetzung von Entzündungsmarkern [1]. Da Tendinopathien dazu neigen, rezidivierend aufzutreten, sind sie für die Patienten belastend. Zu den gerätegestützten Therapiemöglichkeiten gehören heute Elektrotherapie, Schockwellen und die niederenergetische gepulste elektromagnetische Feldtherapie (PEMF).

Die Anwendung der Behandlung mit niederenergetischer PEMF wird derzeit aufgrund der unzureichenden und widersprüchlichen Studienlage nicht empfohlen. Eine Neubewertung auf der Basis neuer Studienergebnisse ist jedoch für die nächste Auflage der deutschen S2k-Leitlinie für Epicondylitis geplant [2]. Die Stärke der in früheren Studien angelegten Magnetfelder reichte von 0,1 mT bis 30 mT [3]. Studien mit hochenergetischen PEMF-Anwendungen liegen noch nicht vor. Auch konnte bisher kein verlässlicher Nachweis der biochemischen Prozesse der PEMF beschrieben werden [4]. Entzündungsparameter wie c‑reaktives Protein (CRP), die im klinischen Alltag häufig und kostengünstig eingesetzt werden, haben in Studien bisher keine Effekte gezeigt. Es konnte jedoch gezeigt werden, dass die HSP70-Konzentration durch PEMF in vitro beeinflussbar ist [5].

Ziel der Studie war es, die Wirkung von hochenergetischen Magnetfeldern auf die Schmerzwahrnehmung bei lokalen Entzündungsprozessen zu untersuchen, die mit Hilfe der Schmerzdruckschwelle (PPT) quantifiziert wird. Zusätzlich untersuchten wir, ob eine Induktion der HSP70- Synthese als Wirkhinweis auf zellbiologischer DNA-Ebene stattfand.

## Methoden

Diese monozentrische klinische Studie wurde in dem Institut für Sportmedizin der Universität Hamburg durchgeführt. Sie wurde als einfach verblindete, randomisiert-kontrollierte Studie (RCT) konzipiert, die aus einer Verum- und einer Placebogruppe (Sham, Scheinbehandlung) mit Patienten mit Tendinopathien in verschiedenen anatomischen Regionen bestand. Die Probanden wurden über Aushänge in Hamburger Kliniken und über das Internet rekrutiert. Für beide Gruppen waren fünf Messzeitpunkte für den primären Endpunkt vorgesehen. Die Teilnehmer waren für die Art der Intervention (Verum vs. Sham) verblindet und aufgrund der Pseudonymisierung war die statistische Analyse ebenfalls verblindet. Während der 7‑tägigen Studie durften keine Schmerzmittel eingenommen werden. Ein Ethikvotum liegt vor (Universität Hamburg, Fakultät für Psychologie und Bewegungswissenschaft – Lokale Ethikkommission, AZ 2022_057) und die Studie wurde beim DRKS (Deutsches Register für Klinische Studien, DRKS00031321) registriert. Die Studie wurde in Übereinstimmung mit der Deklaration von Helsinki für die Forschung mit menschlichen Freiwilligen durchgeführt.

### Stichprobe

**Abb. 1:**
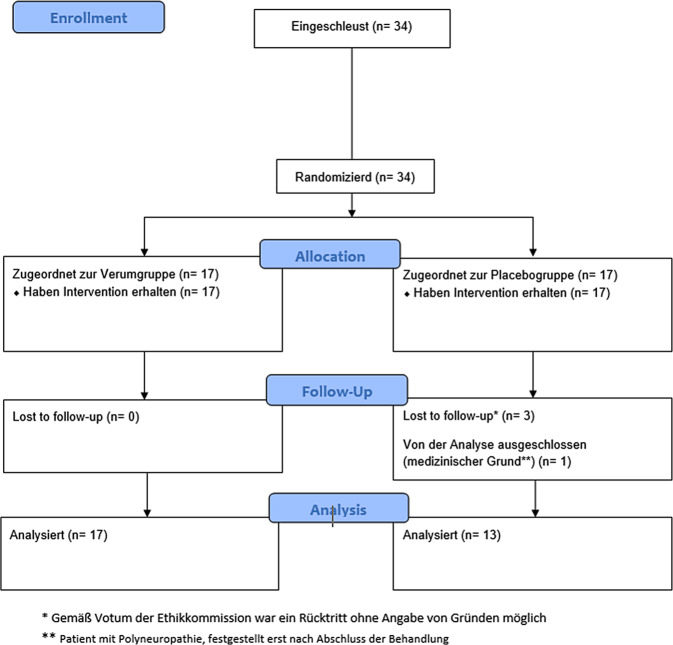
Consort 2010 Flow-Diagramm

A priori wurde eine Power-Analyse zur Stichprobengrößenschätzung für ein Datenmodell mit 2 Gruppen und 5 Messzeitpunkten durchgeführt (G*Power, V.3.1.9.4, Franz Faul, Universität Kiel, Deutschland). Bei einer angestrebten mittleren Effektstärke (f = 0,25) und einer Power (1-β) von 90 % bei einem Signifikanzniveau (α) von 5 % ergab sich eine notwendige Stichprobengröße von 26 Teilnehmern, d. h. 13 Patienten pro Gruppe. In Erwartung eines Drop-outs von mehr als 25 % wurden 34 Probanden rekrutiert und nach dem Zufallsprinzip entweder der Verum- oder der Placebogruppe zugewiesen (Research Randomizer, 4.0) (Abb. 1). Die Patientenrekrutierung erfolgte im Zeitraum vom 01.12.2022 bis 18.03.2023.

Für eine Inklusion war die schriftliche Einwilligung, ein Mindestalter von 18 Jahren und die juristische Geschäftsfähigkeit erforderlich. Es musste eine Tendinopathie mit einem genau lokalisierbaren Schmerzpunkt vorliegen. Einschränkungen der Schmerzwahrnehmung, Implantate, Krebs oder Schwangerschaft galten als Ausschlusskriterien.

In der Verum-Gruppe gab es keine Ausfälle (*n* = 17); in der Placebo-Gruppe schieden jedoch vier Personen aus oder mussten ausgeschlossen werden (*n* = 13) (Abb. 1). Die Daten von 30 Teilnehmern konnten der statistischen Analyse zugeführt werden (Alter 39,8 ± 18,4 Jahre, Körpergröße 176,1 ± 8,6 cm, Körpermaße 72,5 ± 9,6 kg, BMI (Body-Mass-Index) 23,3 ± 2,0 kg/m^2^). Die individuellen Erkrankungen wurden nach ICD 10 (International Classification of Diseases 10th Revision) kodiert (Tab. 1).

**Tab. 1:** Stichprobe

	Verum	± SD	Placebo	± SD
*Alter (Jahren)*	41,5	18,5	37,7	19,4
*Größe (cm)*	175,7	8,9	176,6	8,8
*Gewicht (kg)*	71,9	9,4	73,3	10,5
*BMI (kg/m*^*2*^*)*	23,2	2,2	23,4	2,0
*Anzahl (%)*	17	(56,7)	13	(43,3)
*Frauen (%)*	8 von 17	(47,1)	6 von 13	(46,2)
*ICD 10 Code*				
M62.65 (%)	–	–	1	(7,7)
M67.12 (%)	–	–	1	(7,7)
M67.17 (%)	1	(5,9)	–	–
M70.1 (%)	1	(5,9)	–	–
M72.4 (%)	1	(5,9)	–	–
M75.1 (%)	–	–	1	(7,7)
M76.5 (%)	4	(23,5)	5	(38,5)
M76.6 (%)	3	(17,6)	2	(15,4)
M76.8 (%)	2	(11,8)	1	(7,7)
M77.0 (%)	2	(11,8)	1	(7,7)
M77.1 (%)	2	(11,8)	–	–
M77.9 (%)	1	(5,9)	–	–

Diagnosen: *M62.65* Muskelzerrung; *M67.12* Kontraktur Oberarme; *M67.17* Kontraktur Knöchel/Fuß; *M70.* Bursitis Hand; *M72.4* Plantar fasciitis rechts; *M75.1* Supraspinatussehnensyndrom; *M76.5* Patellarspitzensyndrom; *M76.6* Achillodynie; *76.8* Tendonitis quadriceps; *M77.0* Epicondylitis med.; *M77.1* Epicondylitis lat.; *M77.9* Ansatztendinopathie M. sartorius

### Datenerhebung

**Abb. 2:**
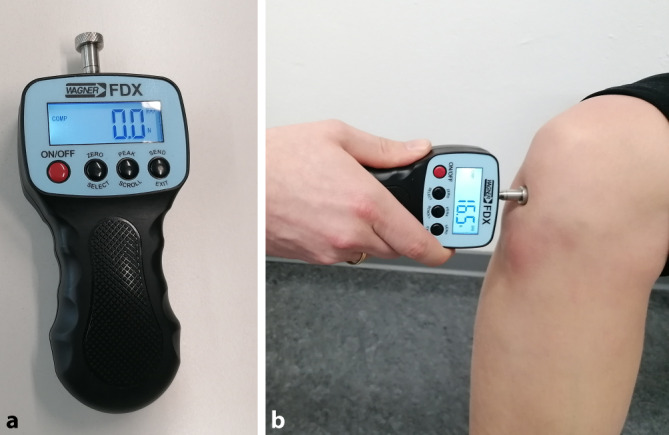
Algometer: **a** Wagner FDX 100 Algometer; **b** Algometer in Benutzung

Der primäre Endpunkt (out-come) war das subjektive Schmerzempfinden, das über die Druckschmerzschwelle (pain pressure threshold, PPT) operationalisiert wurde. Die PPT wurde mit einem Algometer (FDX 100, Wagner Instruments, Greenwich, CT, USA) erfasst (Abb. 2). Diese Methode erwies sich als zuverlässig (Intraklassenkorrelationskoeffizient 0,54–0,95) und wurde bereits in verschiedenen Studien zur Schmerzbeurteilung eingesetzt [6, 7]. Dabei wird der lokalisierte Schmerzpunkt markiert und nach Auflegen einer Druckplatte wird der Anpressdruck kontinuierlich erhöht, bis der Patient Schmerzen angibt. Das Ergebnis wird in Newton $\frac{kgxm}{s^{2}}$ ausgegeben, dokumentiert und der statistischen Analyse zugeführt.

HSP70 wurde als sekundärer Endpunkt bestimmt. HSP70 (66–78 kDA) spielt eine wichtige Rolle bei der Proteinfaltung und dem Proteintransport. Auf diese Weise unterdrückt HSP70 Entzündungsreaktionen, wirkt zellstabilisierend, verringert die Apoptoserate und Degeneration [8]. Für die Analyse verwendeten wir den ELISA-Assay HSP2MAG-63K Kit von Merck Millipore nach dem Protokoll des Herstellers (Merck KGaA 2020). Dazu war es notwendig, das Blut zu zentrifugieren und das Serum abzupipettieren. Das Serum wurde dann bei −20 °C gelagert. Die Proben wurden als Gesamtpacket am Ende der Studie gemeinsam analysiert. Die Analyse erfolgte mit einem Luminex 200 (Merck Millipore).

### Behandlungsintervention

**Abb. 3:**
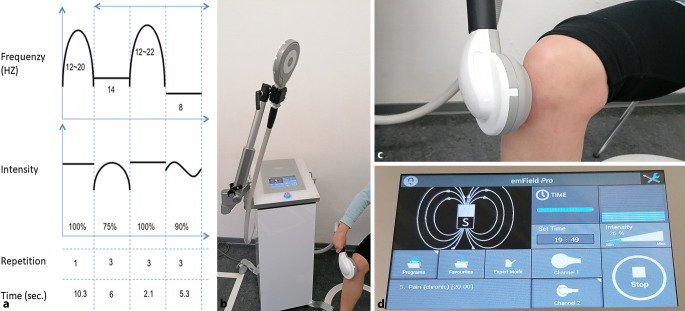
emFieldPro ®: **a** Programm Nr. 5 (Zimmer Medical System); **b** emFieldPro® Magnet-Field-Generator mit zwei Applikatoren; **c** emFieldPro® in Benutzung; **d** Display Einstellungen emFieldPro®

Hochenergetische PEMF wurde mit dem Magnetfeldgenerator emFieldPro® (Zimmer Medical Systems, Neu-Ulm, Deutschland) appliziert (Abb. 3). Der emFieldPro® verfügt über zwei Applikatorköpfe mit unterschiedlichen Durchmessern und erzeugt Magnetfelder mit einer Stärke von bis zu drei Tesla (3 T). Während der Anwendung durchläuft das Gerät verschiedene vorprogrammierte Frequenz- und Intensitätsmuster. Wir wählten ein Programm, das ein Intensitätsniveau von 26 % der maximalen Leistung erzeugt (emFieldPro-Software, Programm Nr. 5). Höhere Energielevel als 26 % wurden in früheren Untersuchungen nicht von allen Probanden toleriert, da sie Schmerzen und starke Muskelzuckungen verursachten. Das hier gewählte Programm erzeugte wiederholt (alle 50,5 Sek.) das in Abb. 3 dargestellte Pulswellenmuster, das eine Leistung von 0,59–0,78 T mit Frequenzen von 8–22 Hz erreichte.

### Experimentelles Protokoll

**Abb. 4:**
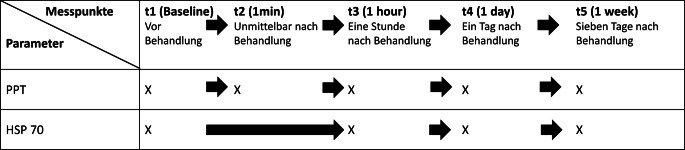
Studiendesign

Nach Überprüfung der Ausschlusskriterien wurde am ersten Tag eine medizinische Untersuchung durchgeführt. Anschließend wurden die anthropometrischen Daten (Tab. 1) erfasst, gefolgt von der ersten PPT-Erhebung und einer Blutentnahme zur Bestimmung der Baseline-Werte des HSP70 (Messzeitpunkt t1). Vor der Anwendung des EmFieldPro®-Magnetfeldgenerators wurden die Probanden beider Gruppen gebeten, elektronische Geräte in einen dafür vorgesehenen Behälter zu legen, und es wurde über mögliches Kribbeln, Muskelzucken oder leichtes Unbehagen aufgeklärt. Die zu behandelnden Regionen wurden entkleidet. Das jeweilige Körperteil wurde in eine neutrale Gelenkposition gebracht. Schließlich wurde der Kopf des Geräts zehn Minuten lang mit Hautkontakt auf die zu behandelnde Stelle aufgesetzt. Die Verumgruppe erhielt eine einzige Sitzung mit magnetischen Impulsen, die vom aktivierten Kopf des Geräts abgegeben wurden, während die Placebogruppe eine Scheinbehandlung (Sham) erhielt, die aus der Applikation eines inaktiven Kopfes bestand, die jedoch durch den räumlich entfernten und aktivierten Applikatorkopf mit den typischen PEMF-Geräuschen begleitet wurde. Nach der 10-minütigen Anwendung wurden die Probanden gebeten, langsam aufzustehen und sich anzuziehen. Anschließend wurden 4 Nachuntersuchungstermine (t2–t5) durchgeführt (Abb. 4). Am Ende des letzten Termins erfolgte die persönliche Benachrichtigung der Teilnehmer über die Gruppenzuordnung und die Auszahlung der Aufwandsentschädigung. Probanden, die ursprünglich der Placebogruppe zugeordnet waren, erhielten die Möglichkeit für eine unmittelbar durchzuführende reale Anwendung.

### Statistische Methoden

Kontinuierliche Daten wurden als Mittelwert (M) und Standardabweichung (SD), bei Illustrationen mit 95 %-Konfidenzintervallen (95 %CI) beschrieben. Im Falle kategorialer Daten (z. B. Geschlecht oder spezifische Diagnose) wurden absolute (*n*) und relative (%) Häufigkeiten beschrieben und Unterschiede in den Stichprobenmerkmalen mittels χ^2^-basierter Statistiken analysiert, einschließlich Cramer’s V (Kontingenzkoeffizient) als Maß für die Effektstärke. Wenn die Normalverteilung nicht bestätigt werden konnte (z. B. für das Alter), wurden die intervallskalierte Daten mit Hilfe nichtparametrischer Verfahren (Mann/Whithney U‑Test) analysiert. Da eine Normalverteilung für Größe, Gewicht, BMI und die abhängigen Variablen (PPT und HSP70) angenommen werden durfte, wurden diese Baseline-Datensätze mit dem Student’s t‑Test auf Gruppenunterschiede geprüft. Die abhängigen Variablen zur Beschreibung der Interventionseffekte wurden mit einer 2‑faktoriellen Varianzanalyse mit Messwiederholung (2-way rANOVA, allgemeines lineares Modell: 2 Gruppen × 5 Zeitpunkte) analysiert. Bei signifikanten Effekten wurden post-hoc die Bonferroni-Prozedur adjustierten multiplen Mittelwertsunterschiede geprüft. Die Anwendungsvoraussetzungen (Normalverteilung, Varianzhomogenität) durften nach Prüfung (Shapiro-Wilk-Test, Levene-Test) angenommen werden. Bei Verletzung der Sphärizitätsannahme (Mauchly-Test) wurden die p‑Werte nach der Greenehouse-Geisser-Korrektur angepasst. Signifikanz wurde bei *p* ≤ 0,05 angenommen. Ein partielles eta^2^ (η^2^ p) von 0,01, 0,06 oder 0,14 deutete auf geringe, moderate, bzw. starke Effekte hin. Die Berechnungen und grafischen Darstellungen wurden mit JASP (V.0.15, JASP Team, Universität Amsterdam, Niederlande) durchgeführt.

## Ergebnisse

In Folge von Randomisierung und Drop-out ergaben sich zur Baseline-Testung keine signifikanten Stichprobenunterschiede (*p* > 0,05) in der Geschlechterverteilung (Verum 47,1 % Frauen vs. Placebo 46,2 %, χ^2^ = 0,002, *p* = 0,961, V = 0,009) und in anthropometrischen Daten (mittlerer Altersunterschied 3,8 Jahre, U = 89,500, *p* = 0,389; mittlerer Größenunterschied 0,91 cm, t = 0,278, *p* = 0,783; mittlerer Gewichtsunterschied 1,425 kg, t = 0,390, *p* = 0,700; mittlerer BMI-Unterschied 0,005 kg/m^2^, t = 0,006, *p* = 0,995) (Tab. 1). Das galt auch für die Häufigkeitsverteilung der Schmerzsyndrome, insbesondere des Patellaspitzensyndroms (M76.5) als möglichem Confounder (χ^2^ = 1,697, *p* = 0,193). Gruppenunterschiede zur Baseline Testung oder im Vergleich zu anderen Diagnosen waren nicht signifikant (t = 0,864, *p* = 0,395), obwohl oberflächlich ein Gruppenmittelwertunterschied von 5,39 N vorlag (M76.5-Fälle 32,01 ± 18,97 N vs. Nicht-M76.5-Fälle 26,63 ± 14,53 N).

Trotz deskriptiv beobachtbarer Unterschiede – insbesondere bei PPT – unterschieden sich die Baselinewerte zwischen Verum- und Placebogruppe für PPT (t = 1,724, *p* = 0,096) und HSP70 (t = 0,646, *p* = 0,524) nicht signifikant.

Die 2‑way rANOVA ergab keine signifikanten Gruppeneffekte (between subjects), d. h. der Faktor Gruppe (Verum vs. Placebo) unter Einbeziehung aller Messzeitpunkte (t1–t5) unterschied sich weder für PPT (*p* = 0,791) noch für HSP70 (*p* = 0,504) signifikant (Tab. 2).

**Tab. 2:** Deskriptive Statistik (M ± SD, 95 %CI) und 2‑way ANOVA Effekte bei wiederholten Messungen

		t1	t2	t3	t4	t5			Gruppe
	Gruppe	base ± *SD*	1 min *±* *SD*	1h *±* *SD*	1 Tag *±* *SD*	1 Woche *±* *SD*	Gruppe	Zeit	× Zeit
*PPT (N)*	Placebo	34,0 ± 17,5	42,1 ± 23,6	43,0 ± 28,7	37,5 ± 19	44,3 ± 24,6	0,791	< 0,001	0,045
	(95 % CI)	(24,3–43,7)	(29,0–55,2)	(27,0–58,9)	(27,0–48,0)	(30,7–58,0)			
	Verum	24,2 ± 13,8	45,8 ± 19,3	45,6 ± 21,6	35,7 ± 17,2	40,2 ± 21,9			
	(95 % CI)	(17,5–30,9)	(36,4 –55,1)	(35,1–56,0)	(27,4–44,0)	(29,6–50,8)			
*HSP70 (ng/ml)*	Placebo	17,8 ± 8,3	–	14,4 ± 5,1	23,3 ± 34,1	10,8 ± 2,9	0,504	0,204	0,386
	(95 % CI)	(13,2–22,5)	–	(11,6–17,2)	(4,4–42,2)	(9,2–12,4)			
	Verum	16,1 ± 5,9	–	16,4 ± 10,8	14,7 ± 12,4	11,7 ± Y8,0			
	(95 % CI)	(13,2–19,1)	–	(10,9–21,8)	(8,5–20,8)	(7,8–15,7)			

Für die PPT ergab die rANOVA jedoch einen signifikanten Zeiteffekt zwischen den Bewertungen vor (t1) und nach der Intervention (t2–t5) (*p* < 0,001), während das nicht für HSP70 galt (*p* = 0,204) (Tab. 2).

Die Veränderungen der PPT nach der Intervention waren für die Verumgruppe signifikant größer als für die Placebogruppe (*p* = 0,045), auch wenn dieser Unterschied lediglich eine moderate Effektstärke aufwies (η^2^ p = 0,013). Post-hoc Analysen separat für die Verumgruppe ergaben, dass die Baseline PPT Werte (t1) signifikant kleiner waren als 1 min (t2: *p* < 0,001), als 1 h (t3: *p* < 0,001), als 1 Tag (t4: *p* = 0,012) und als 1 Woche (t5: *p* < 0,001) nach der Intervention. Dieser Interaktionseffekt war für HSP70 nicht signifikant (*p* = 0,386) (Tab. 2).

**Abb. 5:**
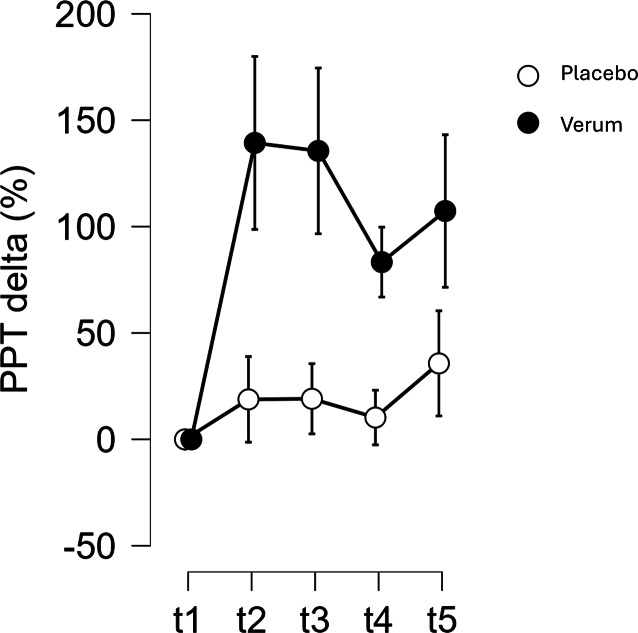
Relative Veränderungen (%) der Druckschmerzschwelle (PPT) von der Baseline (t1) im Verlauf (t2 post-1 min, t3 post-1 h, t4 post-1 day, t5 post-1 week)

Der gruppenspezifische Unterschied in den postinterventionellen PPT Anstiegen kann durch die relativen Veränderungen (%) zu den Zeitpunkten t2–t5 illustriert werden (Abb. 5). Unmittelbar nach der Intervention (t2) erhöhte sich die PPT in der Verumgruppe um 139,3 %, in der Placebogruppe lediglich um 18,8 %. Diese Unterschiede konnten auch für die nachfolgenden Zeitpunkte bestätigt werden (t3: Verum 135,6 % vs. Placebo 19,1 %; t4: Verum 83,3 % vs. Placebo 10,3 %; t5: Verum 107,3 % vs. Placebo 35,7 %) (Abb. 5).

## Diskussion

Ziel dieser klinischen Studie war es, die Wirkung einer einzigen zehnminütigen Sitzung mit hochenergetischem PEMF auf die Schmerzwahrnehmung und HSP70-Veränderungen bei Patienten mit Tendinopathien zu untersuchen. Ohne dass Baseline-Werte zwischen PEMF und Placebogruppe signifikant unterschiedlich waren, fanden wir als Hauptergebnis einen signifikanten Interaktionseffekt für die Schmerzwahrnehmung, der auf eine sofortige und für eine Woche nachhaltige Schmerzlinderung in der Verumgruppe hinwies (83 bis 139 %), während der Placeboeffekt deutlich geringer war (10 bis 36 %). Veränderungen im HSP70 konnten nicht nachgewiesen werden.

Nach Wissen der Autoren ist dieses der erste verblindete RCT mit hochenergetischen PEMF-Anwendungen bei Tendinopathien, in der die Auswirkungen auf Schmerzwahrnehmung und Veränderungen von HSP70 untersucht wurden.

Obwohl keine signifikanten Baseline Gruppenunterschiede beobachtet wurden, soll die Stichprobenheterogenität problematisiert werden. Eine mögliche Ursache für interindividuelle Variationen in der PPT vor der Intervention liegt in der Lokalisation der Druckschmerzwahrnehmung, die z. B. an Knochenpunkten mit nur dünnen Weichteilschichten verstärkt ist. Eine Studie von Melia et al. konnte zeigen, dass diese Spitzendrücke für die Schmerzwahrnehmung relevant sind [9]. Ein Einfluss von Alter, Geschlecht oder anthropometrischen Merkmalen sollte die Ergebnisse jedoch nicht beeinflusst haben [9].

Eine Metaanalyse aus dem Jahr 2015, in der die Wirkung von physiotherapeutischen Übungen untersucht wurde, ergab eine Schmerzreduktion von 50–66 % (Placebo 24,5 %) nach einem Behandlungszyklus von mehreren Sitzungen [10]. Bei unserer Intervention – singuläre 10-minütige PEMF-Sitzung – erhöhte sich die Druckschmerzschwelle unmittelbar (t2) um 138,3 % und war nach einer Woche (t5) immer noch um 107,3 % über dem Baseline Niveau. Der Placeboeffekt ergab eine Reduktion der Schmerzwahrnehmung um 18,8 % (t2), bzw. 35,7 % (t5). Die Placeboeffekte dieser physikalischen Therapieintervention lagen damit im Rahmen früherer klinischer Studien [10, 11].

Die unmittelbaren Effekte müssen exponiert diskutiert werden, da das PEMF-Magnetfeld einen spürbaren elektrischen Induktionsstrom erzeugt, welcher als potenzielle Nebenwirkung einen „elektrotherapeutischen“ Ansatz hätte. Somit sollten elektrotherapeutische Mechanismen als zusätzliche Quelle zur Erklärung der klinischen schmerzlindernden Wirkung in Betracht gezogen werden [12]. In einer Übersichtsarbeit wurden jedoch keine länger anhaltenden signifikanten schmerzlindernden Effekte für Elektrostimulationstherapieansätze berichtet [13].

Dies lässt den Schluss zu, dass die Schmerzlinderung eine Woche nach der hochenergetischen PEMF-Anwendung (107,3 % Schmerzreduktion) in der vorliegenden Untersuchung wahrscheinlich nicht auf die Induktionsstromwirkung zurückzuführen ist.

Es bleibt spekulativ, ob dieser schmerzreduzierenden Effekte bei regelmäßiger Wiederholung der Behandlung stärker ausgefallen wäre. Summationseffekte wurden für mehrere andere therapeutische Anwendungen berichtet. Für TENS-Geräte wurde eine Behandlungsdauer von 4–90 Tagen mit ein bis vier Anwendungen pro Tag untersucht [12]. Bei der Ultraschalltherapie wurden drei bis 15 Anwendungen über vier bis 21 Tage beobachtet [14]. Bei der extrakorporalen Stoßwellentherapie wurden eine bis fünf Anwendungen analysiert [15]. In Studien über niederenergetischen PEMF wurden Behandlungen mit drei (15–20 min pro Behandlung) oder mehr Sitzungen durchgeführt [16, 17]. Im Gegensatz dazu konnten wir mit einer einzigen 10-minütigen Behandlung eine sofortige Schmerzreduktion für bis zu einer Woche zeigen.

Neben den klinischen Auswirkungen auf die Schmerzlinderung wurden verschiedene Signalkaskaden für die Wirkung von Magnetfeldern auf Zellen untersucht. So konnte gezeigt werden, dass die Anwendung eines Magnetfeldes in Zellkulturen eine erhöhte Bindung von Ca2+ an Calmodulin (CaM) bewirkt. Dies führte zu einer Vielzahl von nachgeschalteten Signalwegen, die mit Stoffwechsel, Entzündung, Apoptose, Gefäßtonus und anderen Bereichen zusammenhängen [18, 19]. Eine weitere Wirkung von Magnetfeldern bezieht sich auf die erhöhte Expression von Adenosinrezeptoren (Typ A2A und A3Ars) auf der Zellmembran [20]. Beide Signalkaskaden können jedoch nur in vitro oder durch die Untersuchung von Gewebeproben nachgewiesen werden.

Um die biologischen Vorgänge nach einer Gewebestimulation mit hochenergetischen PEMF besser zu verstehen, wurde HSP70 als unspezifischen Entzündungsparameter bestimmt werden. Die Stimulation von HSP70 durch Magnetfelder wurde in vitro beschrieben [5]. HSP70 wird bereits als Marker für Entzündungsprozesse in-vivo verwendet (z. B. bei akuten Formen der Multiplen Sklerose) [21]. HSP70 wurde für lokale Entzündungsreaktionen bisher jedoch nicht als Entzündungsindikator verwendet. In der vorliegenden Studie beobachteten wir einen Rückgang von HSP70 um durchschnittlich −11,5 % in unserer Verumgruppe und einen Rückgang von durchschnittlich −9 % in der Placebogruppe, wobei weder ein signifikanter Zeiteffekt noch eine signifikante Interaktion nachgewiesen werden konnte (Tab. 2). Daher müssen wir schlussfolgern, dass die hochenergetische PEMF-Anwendung den HSP70-Stoffwechsel in unseren Studien-Setting nicht beeinflusst. Eine hypothetische Vermittlerrolle von HSP70 im Umfeld von Schmerzlinderung und Entzündungsprozessen konnte somit nicht bestätigt werden.

Unsere Studie hat Stärken und Schwächen. Stärken sind sicherlich das verblindete und randomisiert-kontrollierte Studiendesign mit einer eleganten Sham-(Placebo)Behandlung, die Originalität der Forschungsthematik und die apparative Umsetzung der subjektiven Schmerzempfindlichkeitsdiagnostik für lokale Tenderpoints. Die Algometrie ist für genau lokalisierte Tendinopathien wesentlich sensitiver ist als herkömmliche Schmerzskalen.

Die heterogene Stichprobe mit unterschiedlichen Diagnosen bei den inkludierten Schmerzpatienten impliziert jedoch Verzerrungsrisiken. Es kann nicht ausgeschlossen werden, dass verschiedene Tendinopathien auch mit unterschiedlichen Heilungs- oder Schmerzlinderungsmustern assoziiert sind. Dieser Bias könnte unsere Ergebnisse beeinträchtigt haben.

## Ausblick

Zum Nachweis möglicher biologischen Mechanismen sind weitere Arbeiten notwendig. Daneben sollten kumulierte Stimuli (über mehrere Tage) in der zukünftigen Arbeiten evaluiert werden.

## Fazit für die Praxis

- Interaktionseffekt-Analysen ergaben eine signifikante Schmerzreduktion in der Verumgruppe.
- Veränderungen des HSP70-Spiegels konnten nicht beobachtet werden.
- Hochenergetische PEMF-Anwendungen sind wahrscheinlich für eine wirksame Behandlung von Tendinopathien geeignet

## Supplementary Information

Bonferroni-korrigierte post-hoc-Mehrfachvergleiche für den Interaktionseffekt Gruppe x Zeit ESM

## References

[1] Millar NL, Silbernagel KG, Thorborg K et al (2021) Tendinopathy. Nat Rev Dis Primers 7:1. 10.1038/s41572-020-00234-1 33414454 10.1038/s41572-020-00234-1

[2] Tischer T (2019) Leitlinie der Deutschen Gesellschaft für Orthopädie und Orthopädische Chirurgie S2k – Epicondylopathia radialis humeri

[3] Pasek J, Pasek T, Sieroń-Stołtny K et al (2016) Electromagnetic fields in medicine—The state of art. Electromagn Biol Med 35:170–175. 10.3109/15368378.2015.1048549 26192151 10.3109/15368378.2015.1048549

[4] Lee S‑K, Park S, Gimm Y‑M et al (2014) Extremely low frequency magnetic fields induce spermatogenic germ cell apoptosis: possible mechanism. Biomed Res Int 2014:567183. 10.1155/2014/567183 25025060 10.1155/2014/567183 PMC4082851

[5] Rodríguez de la Fuente AO, Alcocer-González JM, Heredia-Rojas AJ et al (2009) Effect of 60 Hz electromagnetic fields on the activity of hsp70 promoter: An in vitro study. Cell Biol Int 33:419–423. 10.1016/j.cellbi.2008.09.014 18957326 10.1016/j.cellbi.2008.09.014

[6] Schneebeli A, Falla D, Clijsen R et al (2020) Myotonometry for the evaluation of Achilles tendon mechanical properties: a reliability and construct validity study. BMJ Open Sport Exerc Med 6:e726. 10.1136/bmjsem-2019-000726 32153987 10.1136/bmjsem-2019-000726 PMC7047478

[7] Gervasi M, Barbieri E, Capparucci I et al (2021) Treatment of Achilles Tendinopathy in Recreational Runners with Peritendinous Hyaluronic Acid Injections: A Viscoelastometric, Functional, and Biochemical Pilot Study. J Clin Med. 10.3390/jcm10071397 33807327 10.3390/jcm10071397 PMC8037202

[8] Gaynora JS, Hagberg S Gurfeinc BT Veterinary applications of pulsed electromagnetic field therapy 10.1016/j.rvsc.2018.05.005 10.1016/j.rvsc.2018.05.005 29775839

[9] Melia M, Geissler B, König J et al (2019) Pressure pain thresholds: Subject factors and the meaning of peak pressures. Eur J Pain 23:167–182. 10.1002/ejp.1298 30076659 10.1002/ejp.1298

[10] Weber C, Thai V, Neuheuser K et al (2015) Efficacy of physical therapy for the treatment of lateral epicondylitis: a meta-analysis. BMC Musculoskelet Disord 16:223. 10.1186/s12891-015-0665-4 26303397 10.1186/s12891-015-0665-4 PMC4549077

[11] Hróbjartsson A, Gøtzsche PC (2010) Placebo interventions for all clinical conditions. Cochrane Database Syst Rev 2010:CD3974. 10.1002/14651858.CD003974.pub3 20091554 10.1002/14651858.CD003974.pub3 PMC7156905

[12] Gibson W, Wand BM, O’Connell NE (2017) Transcutaneous electrical nerve stimulation (TENS) for neuropathic pain in adults. Cochrane Database Syst Rev 9:CD11976. 10.1002/14651858.CD011976.pub2 28905362 10.1002/14651858.CD011976.pub2 PMC6426434

[13] Rushton DN (2002) Electrical stimulation in the treatment of pain. Disabil Rehabil 24:407–415. 10.1080/09638280110108832 12033995 10.1080/09638280110108832

[14] Yalvaç B, Mesci N, Geler Külcü D et al (2018) Comparison of ultrasound and extracorporeal shock wave therapy in lateral epicondylosis. Acta Orthop Traumatol Turc 52:357–362. 10.1016/j.aott.2018.06.004 30497658 10.1016/j.aott.2018.06.004 PMC6204478

[15] van Leeuwen MT, Zwerver J, van den Akker-Scheek I (2009) Extracorporeal shockwave therapy for patellar tendinopathy: a review of the literature. Br J Sports Med 43:163–168. 10.1136/bjsm.2008.050740 18718975 10.1136/bjsm.2008.050740

[16] Giovale M, Novelli L, Persico L et al (2022) Low-energy Pulsed Electromagnetic Field Therapy Reduces Pain in Fibromyalgia: A Randomized Single-blind Controlled Pilot Study. Rheumatol Immunol Res 3:77–83. 10.2478/rir-2022-0013 36465321 10.2478/rir-2022-0013 PMC9524818

[17] Elshiwi AM, Hamada HA, Mosaad D et al (2019) Effect of pulsed electromagnetic field on nonspecific low back pain patients: a randomized controlled trial. Braz J Phys Ther 23:244–249. 10.1016/j.bjpt.2018.08.004 30177406 10.1016/j.bjpt.2018.08.004 PMC6531640

[18] Brighton CT, Wang W, Seldes R et al (2001) Signal transduction in electrically stimulated bone cells. J Bone Joint Surg Am 83:1514–1523. 10.2106/00004623-200110000-00009 11679602 10.2106/00004623-200110000-00009

[19] Pilla A, Fitzsimmons R, Muehsam D et al (2011) Electromagnetic fields as first messenger in biological signaling: Application to calmodulin-dependent signaling in tissue repair. Biochim Biophys Acta 1810:1236–1245. 10.1016/j.bbagen.2011.10.001 22005645 10.1016/j.bbagen.2011.10.001

[20] Varani K, Vincenzi F, Ravani A et al (2017) Adenosine Receptors as a Biological Pathway for the Anti-Inflammatory and Beneficial Effects of Low Frequency Low Energy Pulsed Electromagnetic Fields. Mediators Inflamm 2017:2740963. 10.1155/2017/2740963 28255202 10.1155/2017/2740963 PMC5309410

[21] Cwiklinska H, Mycko MP, Szymanska B et al (2010) Aberrant stress-induced Hsp70 expression in immune cells in multiple sclerosis. J Neurosci Res 88:3102–3110. 10.1002/jnr.22476 20806409 10.1002/jnr.22476

