# Supplementary material for: The effect of electromagnetic fields on tendinopathies: Study on the effect analysis of a singular application of high-energy pulsed electromagnetic fields
Source: Orthopadie (Heidelb). 2024 Aug 22;53(9):668–76. [Article in German] doi: 10.1007/s00132-024-04541-3 (PMC11384642; doi:10.1007/s00132-024-04541-3)
Supplement: Supplementary file 1 — Bonferroni-korrigierte post-hoc-Mehrfachvergleiche für den Interaktionseffekt Gruppe x Zeit ESM [file 132_2024_4541_MOESM1_ESM.pdf]

Bonferroni corrected post-hoc multiple comparisons for the interaction effect group (placebo=0/ verum= 1) x time (t0=pre, t1=post1min, t2=post1h, t3=post1d, t4=post1w)

		mean diff.	95% CI for Mean lower	95% CI for Mean upper	SEM	t	p_{Bonf}	
0, t0	1, t0	9.847	-17.210	36.904	7.673	1.283	1.000	
	0, t1	-8.077	-19.839	3.685	3.514	-2.299	1.000	
	1, t1	-11.765	-38.822	15.293	7.673	-1.533	1.000	
	0, t2	-8.962	-20.724	2.801	3.514	-2.550	0.545	
	1, t2	-11.559	-38.616	15.498	7.673	-1.507	1.000	
	0, t3	-3.500	-15.262	8.262	3.514	-0.996	1.000	
	1, t3	-1.706	-28.763	25.351	7.673	-0.222	1.000	
	0, t4	-10.346	-22.108	1.416	3.514	-2.944	0.177	
	1, t4	-6.206	-33.263	20.851	7.673	-0.809	1.000	
1, t0	0, t1	-17.924	-44.981	9.133	7.673	-2.336	1.000	
	1, t1	-21.612	-31.898	-11.326	3.073	-7.034	< .001	***
	0, t2	-18.809	-45.866	8.249	7.673	-2.451	0.851	
	1, t2	-21.406	-31.692	-11.120	3.073	-6.966	< .001	***
	0, t3	-13.347	-40.404	13.710	7.673	-1.740	1.000	
	1, t3	-11.553	-21.839	-1.267	3.073	-3.760	0.012	*
	0, t4	-20.193	-47.250	6.864	7.673	-2.632	0.548	
	1, t4	-16.053	-26.339	-5.767	3.073	-5.224	< .001	***
0, t1	1, t1	-3.688	-30.745	23.369	7.673	-0.481	1.000	
	0, t2	-0.885	-12.647	10.878	3.514	-0.252	1.000	
	1, t2	-3.482	-30.539	23.575	7.673	-0.454	1.000	
	0, t3	4.577	-7.185	16.339	3.514	1.303	1.000	
	1, t3	6.371	-20.686	33.428	7.673	0.830	1.000	
	0, t4	-2.269	-14.031	9.493	3.514	-0.646	1.000	
	1, t4	1.871	-25.186	28.928	7.673	0.244	1.000	
1, t1	0, t2	2.803	-24.254	29.860	7.673	0.365	1.000	
	1, t2	0.206	-10.080	10.492	3.073	0.067	1.000	
	0, t3	8.265	-18.793	35.322	7.673	1.077	1.000	
	1, t3	10.059	-0.227	20.345	3.073	3.274	0.064	
	0, t4	1.419	-25.639	28.476	7.673	0.185	1.000	
	1, t4	5.559	-4.727	15.845	3.073	1.809	1.000	
0, t2	1, t2	-2.597	-29.654	24.460	7.673	-0.339	1.000	
	0, t3	5.462	-6.301	17.224	3.514	1.554	1.000	
	1, t3	7.256	-19.802	34.313	7.673	0.946	1.000	
	0, t4	-1.385	-13.147	10.378	3.514	-0.394	1.000	
	1, t4	2.756	-24.302	29.813	7.673	0.359	1.000	
1, t2	0, t3	8.059	-18.998	35.116	7.673	1.050	1.000	
	1, t3	9.853	-0.433	20.139	3.073	3.207	0.079	
	0, t4	1.213	-25.845	28.270	7.673	0.158	1.000	
	1, t4	5.353	-4.933	15.639	3.073	1.742	1.000	
0, t3	1, t3	1.794	-25.263	28.851	7.673	0.234	1.000	
	0, t4	-6.846	-18.608	4.916	3.514	-1.948	1.000	
	1, t4	-2.706	-29.763	24.351	7.673	-0.353	1.000	
1, t3	0, t4	-8.640	-35.697	18.417	7.673	-1.126	1.000	
	1, t4	-4.500	-14.786	5.786	3.073	-1.465	1.000	
0, t4	1, t4	4.140	-22.917	31.197	7.673	0.540	1.000	

* $p < .05$, ** $p < .01$, *** $p < .001$

P-value and confidence intervals adjusted for comparing a family of 45 estimates (confidence intervals corrected using the bonferroni method).
